# Targeted siRNA Screens Identify ER-to-Mitochondrial Calcium Exchange in Autophagy and Mitophagy Responses in RPE1 Cells

**DOI:** 10.3390/ijms160613356

**Published:** 2015-06-11

**Authors:** Thomas D. B. MacVicar, Lilith V. J. C. Mannack, Robert M. Lees, Jon D. Lane

**Affiliations:** Cell Biology Laboratories, School of Biochemistry, University of Bristol, Bristol BS81TD, UK; E-Mails: t.macvicar@uni-koeln.de (T.D.B.M.); lilith.mannack@hotmail.co.uk (L.V.J.C.M.); rl14936@bristol.ac.uk (R.M.L.)

**Keywords:** autophagy, mitophagy, Parkin, SIGMAR1, IP3Rs, ATG5, LC3, calcium

## Abstract

Autophagy is an important stress response pathway responsible for the removal and recycling of damaged or redundant cytosolic constituents. Mitochondrial damage triggers selective mitochondrial autophagy (mitophagy), mediated by a variety of response factors including the Pink1/Parkin system. Using human retinal pigment epithelial cells stably expressing autophagy and mitophagy reporters, we have conducted parallel screens of regulators of endoplasmic reticulum (ER) and mitochondrial morphology and function contributing to starvation-induced autophagy and damage-induced mitophagy. These screens identified the ER chaperone and Ca^2+^ flux modulator, sigma non-opioid intracellular receptor 1 (SIGMAR1), as a regulator of autophagosome expansion during starvation. Screens also identified phosphatidyl ethanolamine methyl transferase (PEMT) and the IP3-receptors (IP3Rs) as mediators of Parkin-induced mitophagy. Further experiments suggested that IP3R-mediated transfer of Ca^2+^ from the ER lumen to the mitochondrial matrix via the mitochondrial Ca^2+^ uniporter (MCU) primes mitochondria for mitophagy. Importantly, recruitment of Parkin to damaged mitochondria did not require IP3R-mediated ER-to-mitochondrial Ca^2+^ transfer, but mitochondrial clustering downstream of Parkin recruitment was impaired, suggesting involvement of regulators of mitochondrial dynamics and/or transport. Our data suggest that Ca^2+^ flux between ER and mitochondria at presumed ER/mitochondrial contact sites is needed both for starvation-induced autophagy and for Parkin-mediated mitophagy, further highlighting the importance of inter-organellar communication for effective cellular homeostasis.

## 1. Introduction

Autophagy is a highly conserved catabolic process encompassing distinct pathways for the delivery of cytoplasmic material to the lysosome for degradation and recycling. The best understood form is macroautophagy (herein described simply as “autophagy”), during which material is degraded via the *de novo* assembly, maturation and trafficking of double membrane-bound autophagosomes that fuse with the lysosomes for content degradation and recycling. Cells express a family of dedicated autophagy-related (ATG) gene products that act sequentially following autophagy activation, to initiate and elongate an autophagic isolation membrane that ultimately matures into a functional autophagosome. Autophagy has the capacity to be non-selective or to become highly specific, as is seen in mitophagy, the process through which damaged or redundant mitochondria are degraded through the autophagy pathway [1]. Mitophagy is essential for cellular homeostasis, but poses unique challenges for the cell with respect to the regulation of mitochondrial structural dynamics and bioenergetics control [2]. Significantly, impaired regulation of autophagy—and in particular, mitophagy—can cause cellular functional decline and cell death, resulting in human diseases.

One of the earliest mechanistic steps in autophagy is the initiation of localised signaling events that define the site of autophagosomal isolation membrane nucleation [3]. Both the endoplasmic reticulum (ER) and mitochondria have been implicated as origins for isolation membrane nucleation [4,5,6,7], with Hamasaki *et al.* arguing that the ER-mitochondrial interface is a primary site for autophagosome biogenesis [8]. This suggests that communication between these distinct organelles may be critical for a robust autophagy response, and it is likely that lipid and Ca^2+^ exchange play important regulatory roles [9]. Mitochondrial Ca^2+^ uptake is crucial for the regulation of a variety of physiological functions and its deregulation has been linked to a number of diseases including neurodegenerative disorders [10]. It was postulated some 20 or so years ago that ER and mitochondrial contact is important for regulating Ca^2+^ transfer between the two organelles [11], and we now know that Ca^2+^ exchange and flux is one of the most vital functional features of ER-mitochondrial contact sites. There are four main physiological needs for the regulated and efficient transfer of Ca^2+^ from the ER to the mitochondria. Firstly, mitochondrial bioenergetic control is dependent on mitochondrial Ca^2+^ influx—at least three citric acid cycle dehydrogenases of the mitochondrial matrix are Ca^2+^-dependent [12], while stimulating mitochondrial Ca^2+^ ([Ca^2+^]_mt_) uptake by treating cells with Ca^2+^ mobilizing agonists such as histamine, an inositol-1,4,5-trisphosphate (IP3)-generating agonist, robustly enhances mitochondrial ATP production [13]. Secondly, many reports have identified mitochondria as dynamic physiological buffers for intracellular Ca^2+^ ([Ca^2+^]_i_) [14]. For example, pancreatic acinar cells have been demonstrated to deploy mitochondria as a firewall in order to confine spikes in [Ca^2+^]_i_ to precise sub-cellular locations [15]. Thirdly, a role for Ca^2+^ flux at ER-mitochondrial contact sites is known to be involved in the intracellular apoptotic cascade that occurs via the opening of the mitochondrial permeability transition pore (MPTP) and cytochrome *c* release [16]. Lastly, changes in Ca^2+^ flux at ER-mitochondrial contact sites have been linked to the regulation of mitochondrial movement due to direct Ca^2+^ binding to the EF hands of the mitochondrial GTPase Miro [17,18,19,20].

At the ER, IP3-receptors (IP3Rs) are key Ca^2+^ release channels that populate ER-mitochondrial contact sites [21]. Three isoforms, IP3R1, 2 and 3, have been found in mammalian cells, and these exist in homo- and heterotetrameric conformations comprising alternatively spliced isoforms that vary between tissues [22,23]. Channel opening is primarily stimulated by the binding of the second messenger IP3 [22], although IP3Rs are also regulated by changes in Ca^2+^ [22,24]. Importantly, cytosolic Ca^2+^ has been identified as a key mediator of autophagy, although results have not always been consistent. For example, elevated [Ca^2+^]_i_ promoted autophagy via Ca^2+^/calmodulin-dependent kinase kinase-beta (CaMKKβ)-mediated activation of AMPK [25]. Conversely, lithium treatment, which inhibits IP3R-mediated Ca^2+^ release via sequestration of the IP3 second messenger, induced autophagy in mammalian cells [26]. In addition, siRNA mediated knockdown of IP3R1 and IP3R3 was found to induce autophagy in HeLa cells, as measured by increased GFP-LC3 puncta formation [27], meanwhile autophagy induction has also been recorded after treatment with the potent IP3R competitive antagonist Xestospongin B [27]. Important studies using chicken DT40 cells lacking all 3 IP3R isoforms which can be rescued using channel mutant IP3Rs, suggested that altered baseline autophagy in the absence of IP3Rs is more likely linked to changes in Ca^2+^ regulated mitochondrial bioenergetics [28,29], rather than being due to the physical association between IP3Rs and Beclin-1 that normally occurs at contact sites between mitochondria and the ER [30].

In this study, we have used siRNA to screen for involvement of regulators of ER and mitochondrial structural dynamics and ER-mitochondrial contact to better understand the relationships between ER and mitochondria during starvation mediated autophagy and Parkin-dependent mitophagy. Parkin is an ubiquitin E3 ligase that is recruited to depolarised mitochondria in a PINK1-dependent fashion to trigger rapid and efficient mitophagy [31,32]. Using hTERT-immortalised human retinal pigment epithelial cells (RPE1) stably expressing YFP-Parkin [2], or either mCherry-GFP-LC3B or GFP-ATG5, we have identified several potentially important players in ER-mitochondrial lipid exchange, Ca^2+^ transfer and ER homeostasis in both autophagy and mitophagy.

## 2. Results

### 2.1. An siRNA Screen for Mitochondrial/Endoplasmic Reticulum (ER) Based Mediators of Starvation-Induced Autophagy Identifies SIGMAR1

**Figure 1:**
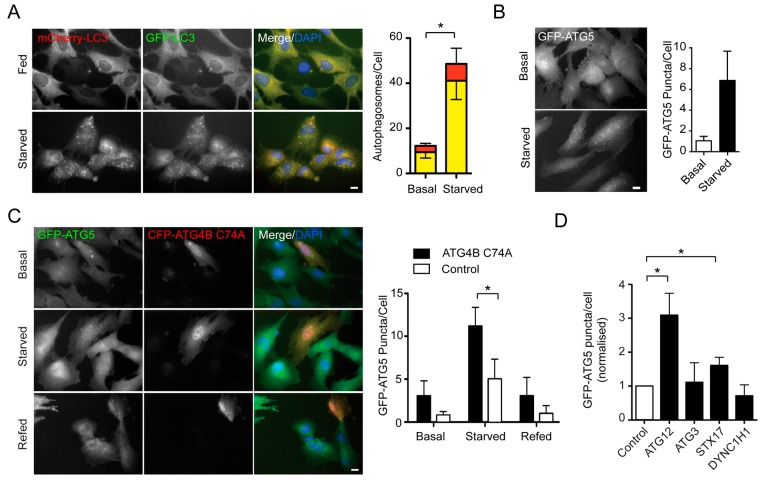
Tandem LC3 RPE1 cells and GFP-ATG5 RPE1 cells are useful reporters for altered autophagosome assembly kinetics. (**A**) Example of a basic assay for starvation-induced autophagosome assembly using RPE1 cells stably expressing mCherry-GFP-LC3B. Images to the left; quantitation of a standard starvation experiment to the right Yellow bars represent red/green autophagosomes; red bars represent red-only autolysosomes (basal = fed; upward error bars are SD of the red-only puncta and downward error bars are SD for green/red puncta; *t*-test * *p* < 0.05 for both elements); (**B**) Example of a basic assay for GFP-ATG5 puncta numbers in fed (basal) and amino acid/growth factor starved RPE1 cells (images to the left; quantitation to the right); (**C**) Increased steady state GFP-ATG5 puncta in cells expressing dominant-negative autophagy mutant, ATG4B C74A (images to the left; quantitation to the right; *t*-test * *p* < 0.5); (**D**) GFP-ATG5 puncta counts in starved cells silenced for ATG12, ATG3, STX17 or DHC1 (*t*-test * *p* < 0.5). Scale bars = 10 µm.

An siRNA screen was designed to evaluate the influence of ER structural dynamics and ER-mitochondrial communication during starvation-induced autophagy. The screen consisted of 61 genes targeted using siRNA SMARTpools (each comprising four siRNA sequences), and the target genes and screen outcomes are listed in Table 1. Two RPE1 cell-lines were generated: one stably expressing tandem mCherry-GFP-LC3B; and the second stably expressing GFP-ATG5, a marker for autophagosome assembly sites. The rationale for the screen was that depletion of any protein involved in autophagosome biogenesis would cause a kinetic delay in assembly, resulting in an accumulation of blocked, GFP-ATG5-positive assembly sites (puncta), and a corresponding reduction in fully-formed LC3-positive autophagosomes. Such a phenotype is diagnostic for stalled autophagosome assembly, and is observed in mouse embryo fibroblasts from the ATG3 knockout mouse [33]. In the RPE1 cell-line, mCherry-GFP-LC3B and GFP-ATG5 puncta numbers increased during amino acid/growth factor starvation (Figure 1A,B). To confirm that a kinetic block in the autophagosome assembly pathway would further increase GFP-ATG5 puncta numbers above the base-line inducible value, cells were transiently transfected with dominant-negative ATG4B C74A, whose expression prevents autophagosome closure [34]. In cells expressing CFP-ATG4B C74A, starvation-induced GFP-ATG5 puncta numbers doubled (Figure 1C). Although this was useful for determining the maximal dynamic range of the GFP-ATG5 response, an siRNA-based positive control was needed for the screen. We therefore tested several possible autophagy-blocking siRNA targets in the GFP-ATG5 RPE1 cell-line, namely: ATG12; syntaxin-17 (STX17); cytoplasmic dynein 1 heavy chain 1 (DYNC1H1); and ATG3 (Figure 1D). The strongest candidate was ATG12; an ubiquitin-like protein that is covalently attached to ATG5 to promote LC3 lipidation upon stimulation. Importantly, ATG5 can localise to unproductive autophagosome assembly sites in the absence of ATG12 [35,36], making this target a useful potential positive control for the stalled autophagosome assembly assay. Correspondingly, depletion of ATG12 caused a significant increase in GFP-ATG5 puncta numbers in starved RPE1 cells (Figure 1D). For the screen, we also included STX17 as a second positive control as this also significantly increased starvation indices GFP-ATG5 puncta numbers, albeit less dramatically (Figure 1D).

**Table 1:** Summary of the autophagy and mitophagy screens.

Gene	Gene ID	siRNA Cat. No	Autophagy Screen			Mitophagy Inhibited?
			ATG5	LC3	Defect?	
*DNM1L*	10059	M-012092-01	0.6	0.7	No	No
*HSPA9*	3313	M-004750-03	1.4	1.05	No	No
*MFF*	56947	M-018261-01	1.7	0.6	No	No
*MARCH5*	54708	M-007001-0	0.65	0.6	No	No
*FIS1*	51024	M-020907-02	0.85	0.3	No	No
*MFN1*	55669	M-010670-01	1.0	0.4	No	No
*MFN2*	9927	M-012961-00	1.0	0.15	No	No
*RHOT1*	55288	D-010365-01	0.85	0.85	No	ND
*RHOT2*	89941	M-008340-01	0.65	0.4	No	ND
*BCAP31*	10134	M-018679-00	1.4	0.1	No	No
*PACS2*	23241	M-022015-01	0.65	0.3	No	No
*TRAK1*	22906	M-020331-02	0.8	0.5	No	ND
*TRAK2*	66008	M-014141-00	1.2	0.4	No	ND
*VAPB*	9217	M-017795-00	1.85	0.35	Yes	No
*STX18*	53407	M-020624-00	1.05	0.05	No	No
*VDAC1*	7416	M-019764-00	0.95	0.25	No	No
*IP3R1*	3708	M-006207-01	0.3	0.85	No	No
*IP3R2*	3709	M-006208-01	0.55	0.55	No	No
*IP3R3*	38710	M-006209-02	0.55	0.8	No	No
*ATF6*	22926	M-009917-01	1.05	0.55	No	No
*EIF3AK3*	9451	M-004883-03	0.55	0.7	No	No
*ERN1*	2081	M-004951-02	0.5	1.1	No	No
*ANK2*	287	M-008417-02	0.65	1.4	No	No
*ATP2A1*	487	M-006113-00	1.4	0.3	No	No
*ATP2A2*	488	M-004082-01	1.9	0.4	Yes	No
*ATP2A3*	489	M-006114-01	0.85	0.75	No	No
*RMDN3*	55177	D-020973-01	0.7	0.35	No	No
*TMBIM6*	7009	M-004118-01	0.9	2.1	No	No
*ESYT1*	23344	M-010652-00	1.3	1.35	No	No
*ESYT2*	57488	M-025231-00	0.8	1.35	No	No
*ESYT3*	83850	M-023602-01	0.55	0.45	No	No
*SIGMAR1*	10280	M-017475-02	2.3	0.2	Yes	No
*PEMT*	10400	D-010392-01	0.85	0.8	No	Yes
*PISD*	23761	M-009548-00	1.6	0.85	No	No
*PTDSS1*	9791	M-008568-00	0.65	0.4	No	No
*PTDSS2*	81490	M-008959-00	0.95	0.7	No	No
*TEX2*	55852	M-017117-00	0.9	1.15	No	No
*ATL2*	64225	M-014047-00	0.65	0.65	No	No
*ATL3*	25923	M-010656-00	0.15	1.35	No	No
*CKAP4*	10970	M-012755-01	1.5	0.25	Yes	No
*KTN1*	3895	M-010605-01	0.55	0.95	No	No
*REEP1*	65055	M-014235-01	1.45	0.75	No	No
*REEP2*	51308	M-021201-00	0.85	0.2	No	No
*REEP3*	221035	M-032301-0	0.85	0.9	No	No
*REEP5*	92840	M-019467-0	0.4	0.3	No	No
*REEP6*	7905	M-015555-00	0.4	0.3	No	No
*RTN1*	6252	M-014138-00	1.4	1.1	No	No
*RTN2*	6253	M-012717-00	0.95	0.35	No	No
*RTN3*	10313	M-020088-00	0.9	1.1	No	No
*RTN4*	57142	M-010721-00	1.1	0.45	No	No
*PSEN2*	5664	M-006018-02	1.1	0.55	No	No
*LRPPRC*	10128	M-018773-00	0.45	0.75	No	No
*MAPRE1*	22919	M-006824-00	1.0	1.3	No	No
*PDZD8*	118987	M-018369-01	0.85	0.4	No	No
*SPAST*	6683	M-014070-02	1.55	0.45	Yes	No
*TCHP*	84260	M-014843-01	1.1	1.1	No	No
*APC*	324	M-003869-01	0.45	0.96	No	No
*BAX*	581	M-003308-03	0.25	0.2	No	No
*BAK1*	578	M-003305-02	1.0	0.25	No	No
*BCL2*	596	M-003307-06	0.3	0.5	No	No
*MAP1S*	55201	M-016881-01	0.55	0.65	No	No
*ATG12*	9140	N/A	2.55	0.15	Yes	ND
*STX17*	55014	10620318/9	1.55	0.7	No	ND
*MEAN*	-	N/A	0.93	0.67	N/A	N/A

For the autophagy screen, entries in grey indicate partial phenotypes; ND: not determined; N/A: not applicable.

**Figure 2:**
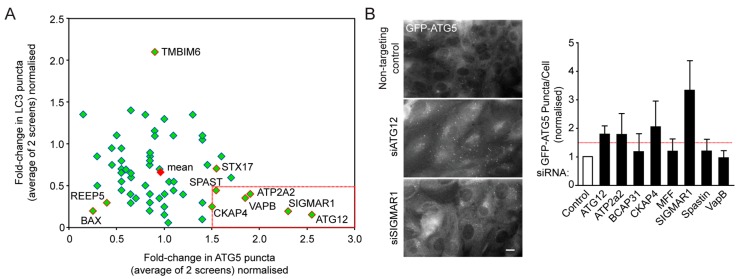

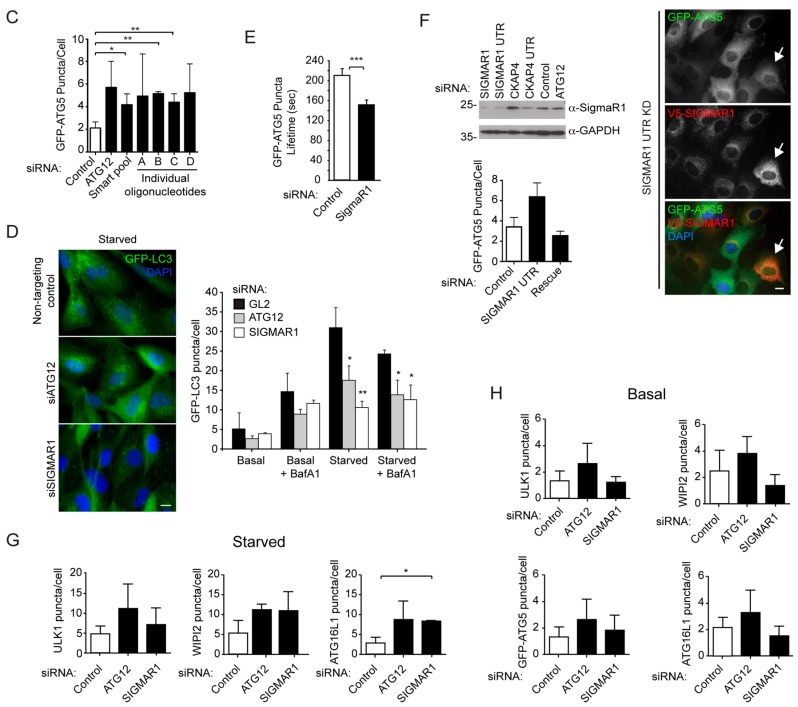
SIGMAR1 suppression blocks autophagy at the isolation membrane expansion/LC3 lipidation stage. (**A**) Pooled data from the combined GFP-ATG5 and GFP-LC3 autophagy screen. Each green diamond represents a single siRNA target. Those with red outlines are identified. A red diamond shows the theoretical mean. The red box represents the cut-off values for positive hits; (**B**) Further characterisation of potential hits using GFP-ATG5 puncta quantitation in starved cells (example images to the left; quantitation to the right); (**C**) Deconvolution of the SIGMAR1 SMARTpool by analysis of GFP-ATG5 puncta in starved cells; (**D**) Reduced LC3 puncta in SIGMAR1-suppressed cells. Example images to the left, quantitation of >400 cells/condition across *n* = 3 experiments are shown to the right; (**E**) Destabilised GFP-ATG5 puncta in SIGMAR1-suppressed cells. Puncta lifetimes measured in GFP-ATG5 RPE1 cells subjected to starvation; (**F**) Rescue of the SIGMAR1 suppression effect using V5-tagged SIGMAR1. An immunoblot of the efficiency of suppression of SIGMAR1 using an oligonucleotide targeting the 3′ untranslated region (UTR), alongside example images of cells suppressed for endogenous SIGMAR1 and expressing V5-SIGMAR1. Quantitation of the rescue based on GFP-ATG5 puncta counts in starved cells is also shown; Arrows indicate an example transfected cell; (**G**,**H**) Measurements of ULK1, WIPI2 and ATG16L1 puncta in SIGMAR1-suppressed cells under basal (**G**) and starvation (H) conditions. *** *p* < 0.001; ** *p* < 0.01; * *p* < 0.05 one-way ANOVA, Tukey’s *post*-test. Scale bars = 10 µm.

A total of 4 autophagy screens were conducted (2 each for mCherry-GFP-LC3 and GFP-ATG5), and those target genes causing a ≥1.5-fold increase in starvation-induced ATG5 puncta numbers and ≥0.5-fold decrease in LC3B puncta numbers with respect to the non-silencing control siRNA were taken on for further validation. The mean outcome of siRNA silencing across all targets was a slight decrease in ATG5 puncta numbers (0.93 normalised to non-targeting control) and a greater decrease in steady state LC3 puncta numbers (0.67), suggesting that many targets reduced the efficiency of autophagosome assembly without necessarily influencing ATG5 kinetics at assembly sites (e.g., MFN1 and 2; Table 1; Figure 2A). Genes whose suppression caused a decrease in both LC3B and ATG5 numbers (suggesting a block in autophagosome assembly upstream of ATG5 recruitment) included BAX, BCL-2, extended synaptotagmin-like protein 3 (ESYT3), phosphofurin acidic cluster sorting protein 2 (PACS2), REEP5, and regulator of microtubule dynamics 3 (RMDN3) (see Table 1; Figure 2A). One candidate, TMBIM6 (also known as BAX inhibitor 1 (BI-1)), was a clear outlier, causing dramatically increased LC3 puncta numbers with unaltered ATG5 levels (Figure 2A). TMBIM6 is an ER Ca^2+^ release channel that has previously been shown to modulate the amplitude of the autophagy response via JNK and IREα [37].

Targets that met pre-determined criteria were further validated by measuring inducible GFP-ATG5 puncta numbers in cells grown on coverslips using conventional (forward) siRNA suppression. These were: ATPase2A2 (ATP2A2); σ-receptor 1 (SIGMAR1); Spastin; and VAMP-associated protein B and C (VAPB). BCAP31 and MFF were also taken forward since their scores were very close to the cut-off point in one of the assays (Table 1; Figure 2B). Figure 2B shows that the observed increase in GFP-ATG5 puncta numbers could not be repeated in cells suppressed for BCAP31, MFF, Spastin or VAPB, so these targets were eliminated. From the screen, the target having the strongest influence on autophagy was SIGMAR1; a ubiquitously expressed and highly conserved mammalian opioid transmembrane receptor located at the ER. The SIGMAR1 lumenal domain binds to the ER chaperone, BiP (Heat Shock 70 kDa Protein 5; HSPA5) to stabilise the IP3Rs in a Ca^2+^-dependent manner, thus linking Ca^2+^ efflux from the ER to the mitochondria with ER proteostasis [38]. To validate the potential role of SIGMAR1 during autophagosome assembly, the siRNA SMARTpool was first deconvolved, whereupon 2 individual oligonucleotides were identified as causing significant increases in steady state GFP-ATG5 puncta numbers in starved RPE1 cells (Figure 2C). SIGMAR1 has previously been linked to autophagy, with data pointing to a role in autophagosome maturation/lysosomal fusion [39]. Interestingly, both our original screens, and additional experiments testing the autophagosome (LC3) response in cells silenced for ATG12 or SIGMAR1 in the absence or presence of Bafilomycin A1 (to block autophagic flux) (Figure 2D), suggested rather that SIGMAR1 is needed during the autophagosome assembly stage in RPE1 cells. We therefore tested whether suppression of SIGMAR1 expression affected GFP-ATG5 dynamics at the assembly site. GFP-ATG5 puncta lifetimes were measured by live-cell imaging of starved control and SIGMAR1-suppressed cells, and this showed that GFP-ATG5 puncta were significantly shorter-lived in cells depleted for SIGMAR1 (Figure 2E). Hence, the increase in steady state GFP-ATG5-positive assembly sites in cells lacking SIGMAR1 might be a compensatory response for reduced autophagosome assembly site stability. Using oligonucleotides targeting the 3’UTR of SIGMAR1, we carried our silencing/rescue experiments using V5-tagged full-length human SIGMAR1 cDNA (kindly provided by Professor Tsung-Ping Su, National Institute of Health, Baltimore, MD, USA). In cells expressing the V5-tagged version of SIGMAR1, GFP-ATG5 puncta numbers returned to control levels, further validating the result of the siRNA screen (Figure 2F).

To try to determine precisely at which point the apparent block in autophagosome assembly was occurring in cells depleted for SIGMAR1, we counted the numbers of punctate structures labelled for staged autophagy markers (namely: ULK1; WIPI2; ATG16L1) in fixed cells under starved and unstarved conditions (Figure 2G,H). These studies suggested that in starved cells, suppression of SIGMAR1 expression mimics the effects of reducing ATG12 expression levels with elevated steady state puncta counts for all markers (although data were only statistically significant for ATG16L1 puncta; Figure 2G). By contrast, and unlike siRNA suppression of ATG12 expression which increased the puncta numbers of all markers in fed conditions (although not statistically significant), none of the markers were elevated above control in SIGMAR1-suppressed cells in the fed state. These data suggest that SIGMAR1 may only be needed during chronic autophagy stimulation, such as occurs during amino acid/growth factor starvation, and is not therefore likely to be a core component of the general autophagosome assembly machinery.

### 2.2. siRNA Screens Identify Lipid Biogenesis and Ca^2+^ Homeostasis Regulators as Mediators of Parkin-Mediated Mitophagy

**Figure 3:**
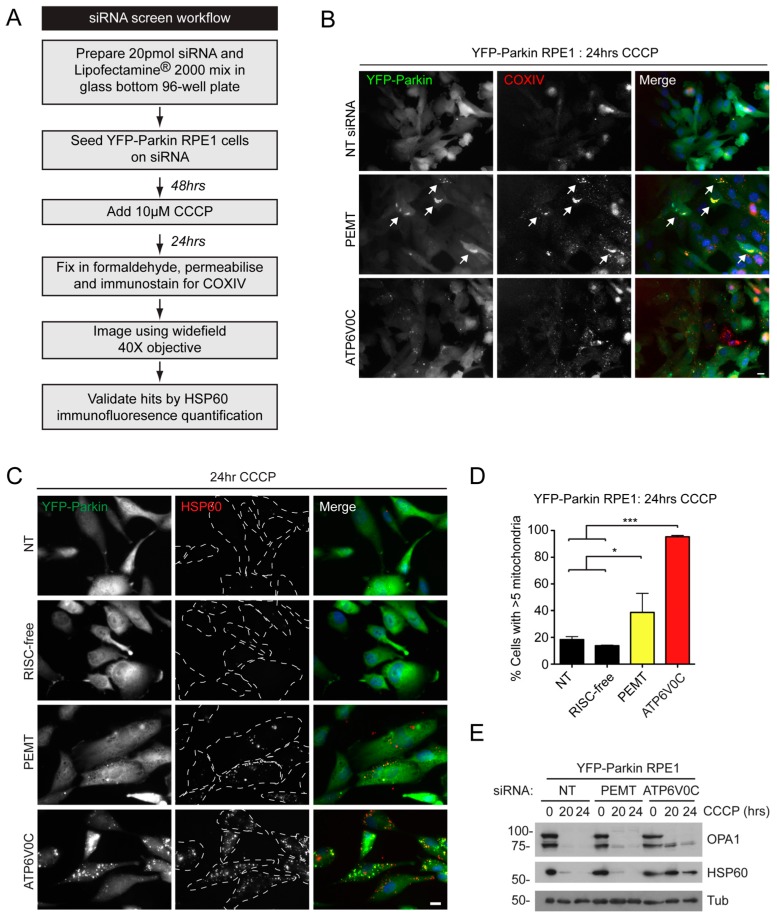
A mitophagy screen identifies phosphatidyl ethanolamine methyl transferase (PEMT). (**A**) Overview of the screen in YFP-Parkin RPE1 cells; (**B**) Example images of COXIV immunostained cells from the 96-well plate mitophagy screen. These examples were form the non-targeting (NT), PEMT and ATP6V0C suppressed wells. Arrows indicate cells with strong mitochondrial retention in the PEMT-treated well (Bar = 10 µm); (**C**) Examples images of mitochondrial content (HSP60 staining) in cells suppressed for the targets shown and treated with CCCP for 24 h (scale bar = 10 µm) (RISC-free siRNA is a control that is not recognized by the RNA-induced silencing complex); (**D**) Quantitation of mitochondrial retention in siRNA-suppressed cells; (**E**) Immunoblot of mitochondrial protein content (OPA1; HSP60) in cells suppressed for PEMT or ATP6V0C, treated with CCCP for the times shown. *** *p* < 0.001; * *p* < 0.05 one-way ANOVA, Tukey’s *post*-test.

We next tested the efficiency of Parkin-mediated mitophagy following siRNA knockdown of the target genes shown in Table 1 using YFP-Parkin expressing RPE1 cells that we had previously validated for mitophagy research [2]. Briefly, YFP-Parkin RPE1 cells were seeded on siRNA in 96 well plates for 48 h, treated with the protonophore 10 µM CCCP for 24 h, fixed then immunostained against mitochondrial COXIV (Figure 3A). The screen was repeated twice and qualitatively analysed for positive hits based on the presence of mitochondrial content after 24 h in CCCP. An initial qualitative screening approach was made possible by the robustness of the mitophagy assay in RPE1 cells ([2]). For a positive control, ATP6V0C—a component of the vacuolar-ATPase (V-ATPase) required for acidification of the lysosome—was silenced (Table 1; Figure 3B). Out of the genes tested, the silencing of only 1 had a positive outcome in both screens. This was identified as the gene coding for PE methyltransferase (PEMT) (Table 1; Figure 3B). To confirm this potential hit, we silenced PEMT alongside 2 negative controls and the positive control, ATP6V0C, in YFP-Parkin RPE1 cells grown in 12-well plates, and measured mitochondrial content following staining for mitochondrial HSP60 by scoring for cells retaining >5 mitochondria (Figure 3C,D), and by immunoblotting (Figure 3E). Although obvious in immunofluorescence-based images, the PEMT mitophagy phenotype was quite subtle, since a strong retention of mitochondrial content in PEMT siRNA knockdown cells was not revealed upon immunoblotting of lysates for HSP60 and OPA1 (Figure 3E).

**Figure 4:**
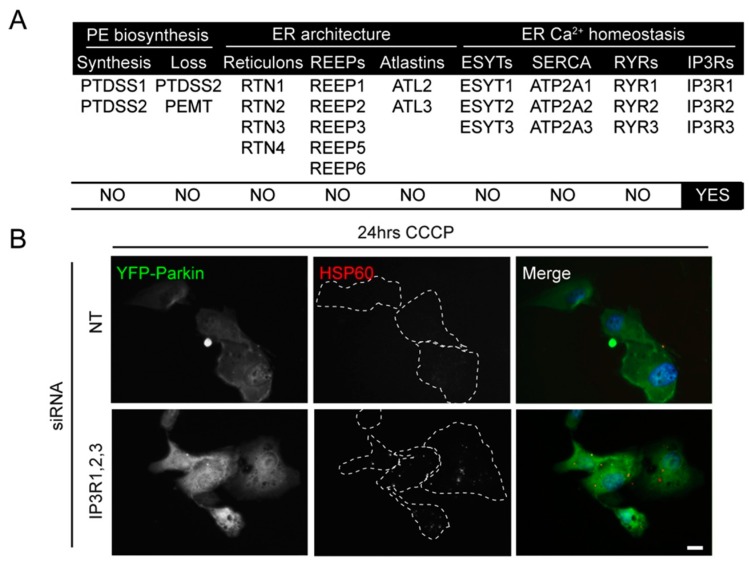

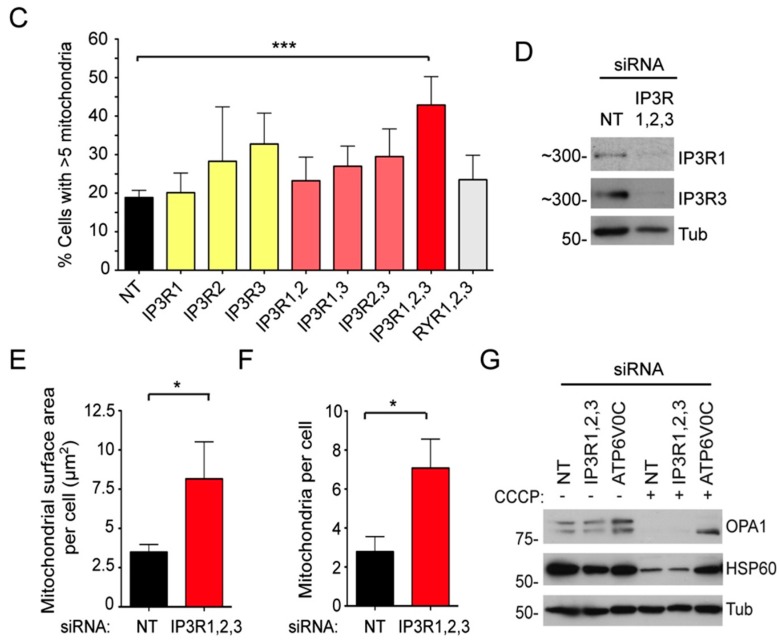
Analysis of potential redundancy in the original screen identifies the IP3Rs. (**A**) Combined siRNA targets and qualitative assessment of mitophagy; (**B**) Example images of mitophagy in YFP-Parkin RPE1 cells suppressed for IP3R1,2,3 (NT = non-targeting control); scale bar = 10 µm. (**C**) Quantitation of mitophagy in YFP-Parkin RPE1 cells suppressed for the targets shown. Cells were treated with CCCP for 24 h. *** *p* < 0.001 one-way ANOVA, Tukey’s *post*-test; (**D**) Immunoblot showing levels of suppression for IP3R1 and IP3R3 in cells suppressed for all 3 IP3Rs; (**E**,**F**) Quantitation of mitophagy in CCCP (24 h) treated cells suppressed for IP3R1,2,3. * *p* < 0.05 *t*-test; (**G**) Immunoblot of mitochondrial proteins (OPA1; HSP60) in YFP-Parkin RPE1 cells suppressed for IP3R1,2,3.

The suppression of other genes involved in the amino-glycerophospholipid biosynthetic pathway, namely PS synthase1/2 (PTDSS1/PTDSS2) and PS decarboxylase (PISD), did not reduce Parkin-mediated mitophagy (Table 1). As both PEMT and PTDSS2 can deplete PE (converting it to PC and PS, respectively) [40], siRNA oligonucleotides targeting each were combined (Figure 4A). In addition, both PTDSS1 and PTDSS2 siRNA pools were combined to further compromise PS synthesis (Figure 4A). A potential for genetic redundancy was spotted in a number of other initial screen targets including the existence of three IP3R and RYR isoforms. With this in mind, further combinations of siRNA pools were devised (Figure 4A), and a second siRNA screen was undertaken. Unexpectedly, no clear mitophagy phenotype was identified following suppression of PEMT and PTDSS2 together (Figure 4A). Likewise, silencing PTDSS1 with PTDSS2, or combinatorial suppression of each member of the reticulon, REEP, and atlastin sub-groups, and the ESYT, SERCA, and ryanodine receptor families, failed to cause detectable mitophagy phenotypes (note that the potential for inefficient siRNA silencing means that it is not possible to say with certainty that any of these are dispensible for Parkin-mediated mitophagy). By contrast, mitophagy was impaired in cells transfected with oligonucleotides targeting all three of the IP3R isoforms (IP3R1,2,3) (Figure 4A,B). To investigate this phenotype further, the IP3R isoforms were deconvolved and mitophagy was quantified by HSP60 immunofluorescence after 24 h CCCP treatment, whereupon a significant increase in the number of YFP-Parkin expressing RPE1 cells with over 5 mitochondria remaining after CCCP treatment was detected only in cells suppressed of all three IP3R isoforms (Figure 4C). Conversely, no mitophagy phenotype was observed in cells suppressed for all three RYR isoforms, suggesting that mitophagy progression is regulated only by IP3R-type Ca^2+^ efflux channel at the ER, and that all 3 isoforms need to be targeted to have an effect (possibly due to functional redundancy). The expression levels of each of the three IP3R isoforms in RPE1 cells is unclear, although all have been reported to be expressed in generic RPE cells [41]. Immunoblotting for isoforms IP3R1 and IPR3 revealed that both are expressed in RPE1 cells, and that pooled IP3R1,2,3 siRNA oligonucleotides suppressed the expression of those isoforms (Figure 4D). Quantitation of mitochondrial area and mitochondrial number confirmed that mitochondria were retained in YFP-Parkin expressing RPE1 cells suppressed for IP3R1,2,3 after 24 h CCCP treatment (Figure 4E,F); however, and in common with the scenario with PEMT silencing (Figure 3D), immunoblotting for HSP60 and OPA1 was not sensitive enough to confirm the clear mitophagy defect observed by microscopy (Figure 4G).

### 2.3. Local Elevated Cytosolic Ca^2+^ Is Needed for Efficient Parkin-Mediated Mitophagy

**Figure 5:**
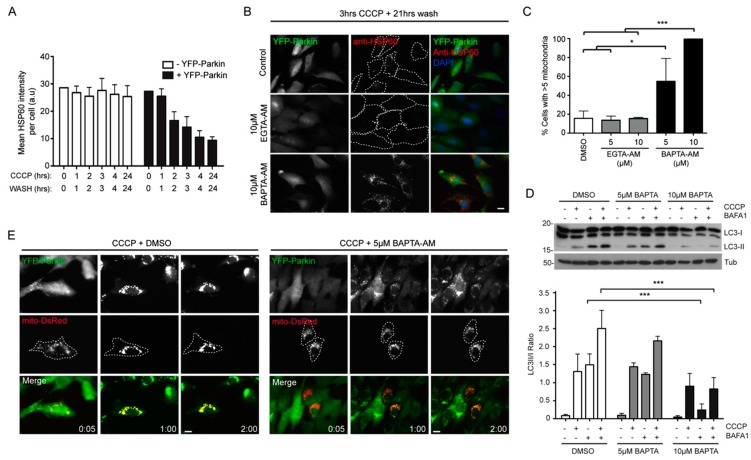
BAPTA-AM blocks mitophagy in YFP-Parkin RPE1 cells. (**A**) Establishing the shortest CCCP treatment for a robust mitophagy response; (**B**) Example images of mitochondrial content in YFP-Parkin RPE1 cells treated with CCCP in the absence or presence of 10 µM EGTA-AM or BAPTA-AM, followed by a 21-h wash; (**C**) Quantitation of mitophagy in cells treated with EGTA-AM or BAPTA-AM using the same treatment-chase procedure (3-h treatment; 21-h wash); (**D**) Measurement of the autophagy (LC3 lipidation) response in cells treated with 5 µM or 10 µM BAPTA-AM in the absence or presence of CCCP; (**E**) Live cell imaging of YFP-Parkin recruitment in RPE1 cells transfected with Mito-dsRed in the absence or presence of 5 µM BAPTA-AM, treated with CCCP. * *p* < 0.05; *** *p* < 0.001 one-way ANOVA, Tukey’s *post*-test. Scale bars = 10 µm.

The defect in mitochondrial clearance in RPE1 cells depleted for all 3 IP3R isoforms may indicate a role for inter-organellar Ca^2+^ signaling in the regulation of Parkin-mediated mitophagy. To test this, the intracellular calcium chelators EGTA-AM and BAPTA-AM were used to establish whether a drop in free [Ca^2+^]_i_ and/or reduced transient exchanges in Ca^2+^ were enough to block mitophagy in this model. EGTA and BAPTA share a similar affinity for Ca^2+^ over other ions like Mg^2+^, but BAPTA has far superior Ca^2+^ binding kinetics than EGTA, since Ca^2+^ associates with BAPTA 50-400 times faster than with EGTA [42]. Importantly, BAPTA can prevent Ca^2+^ signaling across short Ca^2+^ diffusion distances (10–20 nm) whereas EGTA can only have an effect over longer distances (>100 nm) [43]. Hence, Ca^2+^ transfer between the ER and mitochondria at contact sites is likely to be inhibited by BAPTA but not EGTA. Parkin-mediated mitophagy was therefore examined in cells treated with equal concentrations of BAPTA-AM and EGTA-AM. To minimize the exposure length of cells treated with BAPTA-AM or EGTA-AM, an optimization experiment was first performed to determine the minimum length of CCCP treatment sufficient to trigger robust mitophagy in YFP-Parkin RPE1 cells (Figure 5A). Accordingly, the Parkin mitophagy assay was adapted so that cells were treated with CCCP in the presence of Ca^2+^ chelators for only 3 h before the media was replaced with fresh, toxin-free growth media. Cells were then incubated for a further 21 h (*i.e.*, 24 h in total) prior to fixation and immunostaining with a nti-HSP60 antiserum. Under these conditions, treatment of YFP-Parkin RPE1 cells with 5 or 10 µM EGTA-AM had no effect on mitophagy progression, whereas treatment with 5 or 10 µM BAPTA-AM blocked CCCP-induced mitophagy in a concentration dependent manner (Figure 5B,C). These observations imply that transient and local Ca^2+^ spikes might be needed for mitophagy, possibly driven by IP3R-mediated transfer of Ca^2+^ between the ER and mitochondria. To test whether the very strong inhibition of mitophagy after BAPTA-AM treatment was due to a blunted autophagy response to CCCP, LC3 lipidation was quantified in YFP-Parkin RPE1 cells pretreated with 5 or 10 μM BAPTA-AM (Figure 5D). No significant differences in autophagy induction or flux were detected between control and 5 μM BAPTA-AM treated cells; however, autophagic flux was markedly reduced in 10 μM BAPTA-AM pretreated cells (because Bafilomycin A1 treatment did not enhance LC3 processing in the presence or absence of CCCP) (Figure 5D). Together, these results suggest that the strong mitophagy block observed in YFP-Parkin expressing RPE1 cells treated with 10 µM BAPTA-AM is due to impaired autophagic flux, but that at the lower (5 µM) concentration, other factors are responsible. High concentrations of BAPTA-AM (40 µM) also inhibit DRP1 mediated fission in response to CCCP via suppression of Ca^2+^-dependent calcineurin-mediated dephosphorylation of DRP1-Ser637 [44], meaning that altered mitochondrial dynamics might play a contributory role. To pinpoint at which point the Parkin pathway is susceptible to 5 µM BAPTA-AM treatment, live-cell imaging of YFP-Parkin RPE1 cells was conducted (Figure 5E). Importantly, both mitochondrial fragmentation and YFP-Parkin recruitment were found to occur with similar kinetics in both DMSO and 5 µM BAPTA-AM pre-treated cells exposed to CCCP. Interestingly, however, the characteristic perinuclear clustering of Parkin-decorated mitochondria was reduced in the presence of the Ca^2+^ chelator (Figure 5E).

**Figure 6:**
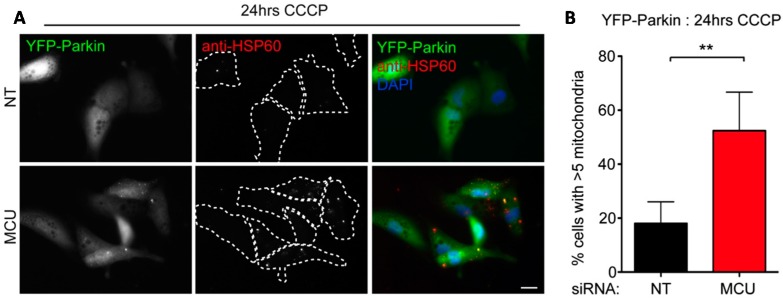
Silencing the mitochondrial Ca^2+^ uniporter (MCU) inhibits Parkin-mediated mitophagy. Example images (**A**) and quantitation (**B**) of mitochondrial content in YFP-Parkin RPE1 cells suppressed for MCU and treated with CCCP for 24 h. ** *p* < 0.01, *t*-test. Scale bar = 10 µm.

The above studies suggested that Ca^2+^ transfer from ER to mitochondria might be a key step in the acute mitophagy response, so in order to test whether uptake of Ca^2+^ into mitochondria influences mitophagy we focused on the mitochondrial Ca^2+^ uniporter (MCU [45]). MCU silencing abolishes the increases in basal [Ca^2+^]_mt_ that occur in cells simultaneously depleted of the [Ca^2+^]_mt_ “gatekeeper” MICU1 [46], and MCU overexpression causes measurable increases in basal [Ca^2+^]_mt_ uptake [47]. In our study, YFP-Parkin RPE1 cells were treated with MCU targeting siRNA for 48hrs before being challenged with 10 µM CCCP for 24 h. Silenced cells demonstrated a reduced mitophagy phenotype which was comparable with that recorded in IP3R1,2,3 suppressed cells (Figure 6). This suggests that reduced [Ca^2+^]_mt_ may have an inhibitory effect on the progression of Parkin-mediated mitophagy. As [Ca^2+^]_mt_ uptake relies on an intact mtΔΨ, which is rapidly dissipated in presence of CCCP, it is possible that altered steady state mitochondrial Ca^2+^ import negatively predisposes mitochondria to mitophagy.

**Figure 7:**
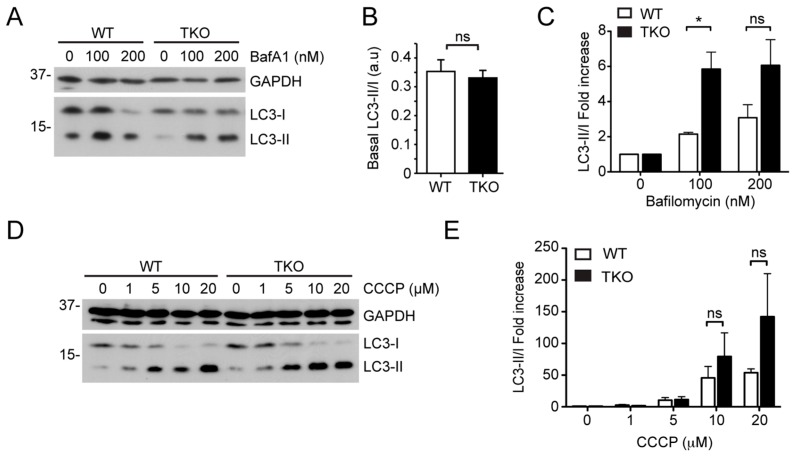

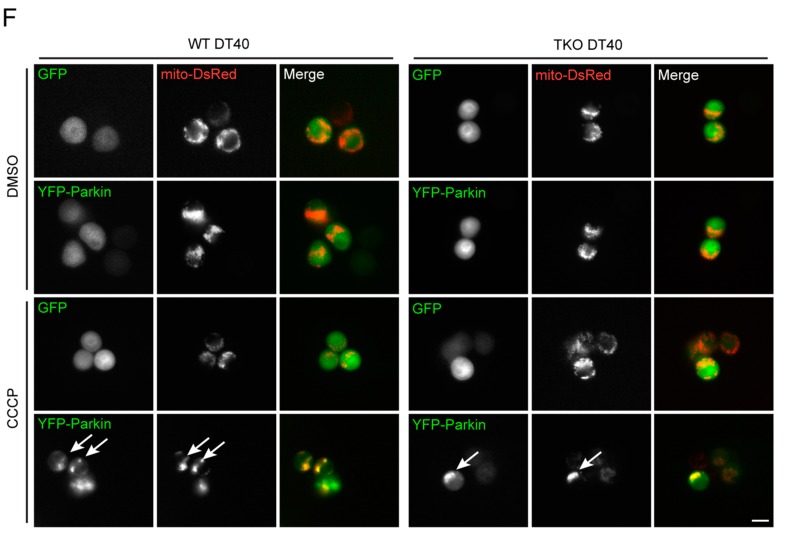
Effect of IP3R depletion on LC3 lipidation and Parkin recruitment to damaged mitochondria. (**A**) Immunoblot of LC3 lipidation in WT and TKO-DT40 cell lysates treated with Bafilomycin A1 for 2 h; (**B**) Quantitation of basal LC3 lipidation in WT and TKO-DT40 cells (*n* = 3; means ± SD; ns = not significant, *t*-test); (**C**) Quantitation of LC3 lipidation in WT and TKO-DT40 cells treated with Bafilomycin A1. (*n* = 3; means ± SD; * *p* < 0.05 2-way ANOVA Bonferroni *post*-test); (**D**) Immunoblot and (**E**) quantitation of LC3 lipidation in WT and TKO-DT40 cell lysates treated with increasing concentrations of CCCP for 1 h (*n* = 3; means ± SD); (**F**) YFP-Parkin recruitment to damaged (CCCP-treated) mitochondria in WT and TKO-DT40 cells co-expressing Mito-dsRed (scale bar = 10 µm). Arrows indicate cells with clustered, Parkin-decorated mitochondria.

ER-to-mitochondrial Ca^2+^ signaling may play differing roles during damage induced mitophagy and basal and starvation-induced autophagy. The inhibition of ER Ca^2+^ release and mitochondrial Ca^2+^ uptake by pharmacological and genetic suppression of IP3R and MCU respectively has been shown to induce basal autophagy [27,28,29,46]. By contrast, IP3R mediated Ca^2+^ signaling has been shown to be essential for mTOR-controlled autophagy responses to nutrient starvation and rapamycin treatment [48,49]. To test whether differing autophagy responses in the absence or presence of IP3Rs might influence Parkin-mediated mitophagy, we used IP3R triple knockout (TKO)-DT40 cells as previously described [28,29,50]. Immunoblotting for LC3 processing revealed that the relative basal levels of lipidated LC3II were similar in wild type DT40 (WT-DT40) and TKO-DT40 cells (Figure 7A,B). This differs from the study of Cardenas *et al.* which described a greater than two-fold increase in the LC3II:LC3I ratio between DT40 genotypes [28]. We also measured autophagic flux in WT- and TKO-DT40 cell by treating them with Bafilomycin A1 for 2 h (Figure 7A,C). As previously reported [29], no inhibition of autophagic flux was detected in the absence of IP3R activity, since TKO-DT40s exhibited a greater increase in LC3II:LC3I ratio after treatment with Bafilomycin A1 than their wild type counterparts (Figure 7A,C). Finally, the autophagy response to varying concentrations of CCCP treatment for 1hr was measured, whereupon a strong (but not statistically significant) enhancement in LC3II levels was detected in CCCP treated TKO-DT40 cells as compared to WT-DT40 cells (Figure 7E). These results suggest that there is a greater rate of basal autophagic flux in untreated TKO-DT40s, and that cells lacking IP3Rs mount a particularly strong stress-induced autophagy response. We next attempted to use the TKO-DT40 cell line to study mitophagy in an IP3R-null background. WT-DT40 and TKO-DT40 cells were co-transfected with mito-DsRed and human YFP-Parkin (or EGFP as a control), and treated cells with 10 µM CCCP. Live-cell imaging revealed that 1 h CCCP treatment was sufficient to trigger YFP-Parkin translocation to the mitochondria in both WT-DT40 and TKO-DT40 cells (Figure 7F), confirming that IP3R function is not required for Parkin recruitment. Unfortunately, we were unable to detect clear evidence of mitophagy in either cell-type, despite the characteristic perinuclear clustering Parkin-decorated mitochondria (Figure 7F), meaning that this system could not be used to study the influence of IP3R function during downstream mitophagy steps.

## 3. Discussion

The autophagy and mitophagy pathways play pivotal roles in the maintenance of cellular homeostasis, and are upregulated in response to diverse cellular challenges. Common molecular pathways regulate autophagosome biogenesis during starvation-mediated autophagy (largely unselective) and during stress-induced mitophagy (highly selective); however, additional proteins that target the assembly of the nascent autophagosome around a mitochondrion are required (e.g., Parkin, Nix, FUNDC1 in mammalian cells (see [1])). The finding that the early autophagosome assembly machinery is focused at ER-mitochondrial contact sites, and that stress-induced autophagosomes originate at or close to these sites [8], supports a persuasive argument that ER and mitochondrial homeostasis combine to coordinate both stress-induced autophagy and mitophagy responses to drive the selective engulfment of mitochondria that are in physical contact with the ER. How cells remain capable of mounting a robust autophagy response during nutrient starvation without superfluously depleting their pools of metabolically active mitochondria has been the subject of some discussion, with altered mitochondrial dynamics to favor network fusion emerging as a credible explanation (see [51,52]). Most notably, in cultured cells that have been forced to use their mitochondria for ATP generation (in galactose media), mitophagy is efficiently blocked by the prevention of mitochondrial fission activities at the inner and outer mitochondrial membranes [2]. Whether a rudimentary size-exclusion phenomenon is sufficient to provide control over the mitophagy pathway *in vivo* remains largely untested, and might not extend to specialized cells such as adult cardiomyocytes that display poorly interconnected mitochondria with reduced dynamic properties [53]. To further examine the potential relationships between molecules acting at ER-mitochondrial contact sites, proteins involved in ER/mitochondrial shaping and dynamics, and the autophagy/mitophagy machineries, we carried out parallel siRNA-based screens the autophagy and mitophagy responses in human RPE1 cells. This approach has provided evidence that ER-mitochondrial communication, and in particular Ca^2+^ transfer between these organelles, is important for the regulation of both starvation-induced autophagy and Parkin-mediated mitophagy. Using RPE1 cells stably expressing markers for autophagosomes (mCherry-GFP-LC3B) or autophagosome assembly site (GFP-ATG5), we identified several candidate proteins that influence the autophagosome expansion/LC3 lipidation stage of autophagosome biogenesis. The strongest candidate was SIGMAR1—an ER resident protein with roles in Ca^2+^ signaling and the unfolded protein response. Meanwhile, the initial Parkin-mediated mitophagy screen identified PEMT—a regulator of lipid biosynthesis—as being required for a robust mitophagy response in CCCP-treated cells, but further screens accounting for redundancy in some of the siRNA target sub-families, also suggested a role for IP3Rs. These findings highlight the likely involvement of Ca^2+^ signaling, although, intriguingly, the different screens identified different key players suggestive of differential requirements for Ca^2+^ in autophagy/mitophagy responses.

Ca^2+^ signaling plays prominent roles in diverse core cellular processes, so it is perhaps not surprising that disturbances in the homeostatic balance of cytosolic and organellar [Ca^2+^] influences the efficiency of the autophagy response. Indeed, numerous studies have implicated Ca^2+^ signaling in autophagy regulation, although a unifying model for how Ca^2+^ modulates the autophagy pathway is currently lacking [54]. The primary candidate we identified in the starvation-induced autophagy screen—SIGMAR1—is interesting because it acts at the interface between ER proteostasis and ER-to-mitochondrial Ca^2+^ signaling through its capacity to interact with and/or regulate both ER luminal BiP and the IP3Rs [38]. A recent independent study showed that SIGMAR1 regulates late phases of autophagosome maturation (autophagic flux), through its suggested roles in Ca^2+^ homeostasis and endomembrane organisation/function [39], although, our analysis of autophagosome assembly site markers point instead to a role at a much earlier stage: in our hands, RPE1 cells suppressed for SIGMAR1 displayed increased steady state numbers of autophagosome assembly sites were recorded during starvation, meanwhile GFP-ATG5-positive puncta in SIGMAR1 silenced cells exhibited a reduced lifetime. Importantly, the abnormal steady state autophagosome assembly site marker quantities recorded in SIGMAR1 cells was not seen in fed cells, although silencing ATG12 in this context did cause and elevation of early autophagosome assembly site markers. One possible interpretation of this is that SIGMAR1 only plays a role in autophagosome assembly in starved cells experiencing chronic autophagy induction. It is possible that the increased/prolonged demand for autophagosome assembly during starvation necessitates the involvement of additional factors to mobile further membrane supplies. Hence, the differences between our findings and the findings of Vollrath *et al.* [39] might simply relate to cell-type variations in membrane supply to the autophagy machinery, although this will need to be further tested.

Extending the screen to the YFP-Parkin RPE1 mitophagy assay revealed that the silencing of all three IP3R isoforms perturbs the efficiency of mitochondrial degradation in response to CCCP treatment. IP3Rs control Ca^2+^ exchange between the ER and mitochondria, and our subsequent experiments suggested that such Ca^2+^ flux might have a bearing on the progression of mitophagy. Silencing the MCU mirrored the mitophagy phenotype exhibited in cells suppressed for IP3R1,2,3, and the fast-kinetic quenching of [Ca^2+^]_i_ by BAPTA strongly inhibited Parkin-mediated mitophagy. More work will be needed to dissect the roles played by Ca^2+^ signaling in mitophagy, and as a first step, accurate measurements of [Ca^2+^]_i_ and [Ca^2+^]_mt_ changes in response to various mitophagy inductions would be beneficial. The inhibition of mitophagy in MCU siRNA-suppressed cells implies that [Ca^2+^]_mt_ levels might be particularly important, but how so remains unclear. Basal levels of [Ca^2+^]_mt_ are normally very low in non-stimulated cells (sub 1 µM), and may be minimal in bioenergetically inactive cells [13], arguing against acute release of mitochondrial Ca^2+^ being a trigger for downstream mitophagy processes. Interestingly, overexpression of Parkin has recently been demonstrated to promote ER-mitochondrial Ca^2+^ transfer in response to IP3 generating stimuli, but the basal concentration of [Ca^2+^]_mt_ did not appear to change in that study [55]. Extensive mitochondrial depolarization by 10 µM CCCP treatment causes a substantial (~250%) increase in [Ca^2+^]_i_ via the release of Ca^2+^ from mitochondria and other intracellular stores [56,57]. Given the relatively low resting [Ca^2+^]_mt_ in unstimulated cells, the relative contributions of [Ca^2+^]_mt_ to the elevated [Ca^2+^]_i_ observed following CCCP treatment in the absence or presence of IP3R/MCU activities are likely to be minimal (although transient spikes of [Ca^2+^]_i_ local to damaged mitochondria might nevertheless be important).

Whether physical tethering between the ER and mitochondria is needed for Parkin-mediated mitophagy has not yet been formally demonstrated. It is possible that our IP3R silencing approach disrupted the physical connection between the ER and mitochondria, thereby impacting on the kinetics of mitophagy through reduced physical coupling. Arguing against this scenario are the data in our initial screen showing that knockdown of other known tethering factors such as GRP75 and MFN2 did not cause a detectable mitophagy defect. Indeed, others have already shown that the progression of YFP-Parkin-mediated mitophagy is completely normal in MFN2^−/−^/MFN1^−/−^ MEFs [58]. However, given that the depletion of GRP75 and MFN2 also reduces Ca^2+^ exchange between the ER and mitochondria [59,60], it would be worthwhile to compare as to what extent mitochondrial Ca^2+^ homeostasis is perturbed upon the loss of different ER-mitochondrial tethers. Rescuing the mitophagy phenotype associated with IP3R1,2,3 knockdown by expression of functional and channel dead mutant forms of the IP3Rs will help differentiate between the roles of ER-mitochondrial tethering and ER-mitochondrial Ca^2+^ transfer in the mechanistic control of mitophagy. One way in which IP3R might normally influence basal autophagy is through interactions with Beclin-1 and Bcl-2 [30]. Beclin-1 co-immunoprecipitates with IP3R in a BCL-2-dependent manner [30]. Importantly, Xestospongin B treatment was shown to disrupt the interaction between Beclin-1 and the IP3R/BCL-2 complex, thus releasing Beclin-1 to induce autophagy [30]. Two studies in IP3R TKO-DT40 cells highlighted the roles played by IP3R in autophagy suppression, but these strongly argued that this is dependent on [Ca^2+^]_mt_ uptake [28,29]. TKO-DT40 cells lacking all three IP3R isoforms had elevated levels of basal autophagy that could be abrogated by expression of a functional IP3R isoform but not by expression of a channel dead mutant [28]. Since the channel mutant can still bind Beclin-1 [28], and no change in Beclin-1 interaction with BCL-2 was detected in TKO-DT40s [29], it was concluded that autophagy induction in TKO-DT40 cells is independent of the IP3R-Beclin-1 association (and instead depended on an elevation in the AMP:ATP ratio and AMPK activation). Surprisingly, our analysis of LC3 lipidation suggested similar basal autophagy levels in WT-DT40 and TKO-DT40 cells, although the latter exhibited greater autophagic flux that probably counter-balanced the elevated basal rate of LC3 lipidation.

Our independent starvation-induced autophagy and Parkin-mediated mitophagy screens have highlighted the involvement of Ca^2+^ signaling in autophagy/mitophagy. That the two primary candidates from either screen—SIGMAR1 and the IP3Rs—cooperate to control ER-to-mitochondrial Ca^2+^ exchange in healthy cells is intriguing, and argues that efforts to understand how this relationship is influenced by cellular stress would be worthwhile. Furthermore, the MCU silencing data might suggest the potential for Ca^2+^-based mitochondrial priming as a prerequisite for mitophagy. In this scenario, mitochondria actively importing Ca^2+^ via the MCU would be predisposed to mitophagy, perhaps due to their greater bioenergetics capabilities or their precise location with respect to the ER Ca^2+^ exchange hub where autophagy regulators also concentrate [8]. Further work will be needed to test this idea, and to decipher the relative requirements for Ca^2+^ signaling during autophagy in different cell-types and cellular stress scenarios.

## 4. Experimental Section

### 4.1. Chemicals and Antibodies

Unless otherwise indicated, all chemicals were obtained from Sigma (Gillingham, UK). Bafilomycin A1 (VIVA Bioscience, Exeter, UK) was prepared in DMSO and was used at a final concentration of 200 nM. The following antibodies were used: anti-ATG16L1 (PM040, MBL, Woburn, MA, USA); anti-COXIV subunit I (Ab14705, Abcam, Cambridge, UK); anti-GAPDH (G8795); anti-HSP60 (H4149); anti-IP3R1 (Abcam, ab5804); anti-IP3R3 (610312, BD Biosciences, San Jose, CA, USA); anti-LC3A/B (L8918); anti-LC3B (L7543); anti-LC3B MBL (PM036); anti-OPA1 (612607, BD Biosciences); anti-SIGMAR1 (Ab53852, Abcam); anti-α-tubulin (T5168); anti-ULK1 (Sc-33182, Santa Cruz Biotechnology, Dalas, TX, USA); anti-V5 tag (R960-25, Invitrogen, Paisley, UK); anti-WIPI2 (MCA5780GA, AbD Serotech, Kidlington, UK).

### 4.2. Cell Culture and Cell-Lines

RPE1 cells were grown in Dulbecco’s Modified Eagles Medium (DMEM) F12 HAM with 3.15 g/L glucose, 2.5 mM l-glutamine (Sigma #D8062) supplemented with 10% FBS (Gibco, Life Technologies, Paisley, UK) at 37 °C in a humidified incubator in the presence of 5% CO_2_. Human embryonic kidney cells containing the SV40 large T antigen (simian vacuolating virus 40 TAg) (ATCC) were grown DMEM with 4.5 g/L glucose (high glucose) supplemented with 10% FBS and 2 mM l-glutamine. RPE1 cells stably expressing YFP-Parkin were described in [2]. DT40 cells were obtained from the Riken Cell Bank, Tsukuba, Ibaraki, Japan. These were grown in RPMI1640 media supplemented with 10% FBS, 1% chicken serum and 50 µM β-mercaptoethanol. Cells were starved for 1 h at 37 °C in a humidified incubator with 5% CO_2_ in a starvation media (140 mM NaCl, 1 mM CaCl_2_, 1 mM MgCl_2_, 5 mM glucose and 20 mM HEPES at pH 7.4 supplemented with 1% (*w*/*v*) fresh BSA) [61].

### 4.3. Transfection and Viral Transduction

DT40 cells were transfected using the next-generation electroporation based Neon™ Transfection System (Invitrogen) according to the manufacturer’s instructions. Cells were resuspended in Buffer R at 1 × 10^8^ cells/mL. 10 μL of the cell suspension was mixed with 2 µg high-quality DNA. A 10 μL Neon™ tip and Neon™ Pipette were used to transfer the cell/DNA mix to the benchtop Neon™ tube containing 3 mL electrolytic buffer. Cell specific electroporation parameters were: pulse voltage 1435 V; pulse width 30 ms; pulse number 1). After electroporation, 1 × 10^6^ of transfected DT40 cells were plated per 35 mm dish. The LentiX™ HTX packaging system (Clontech, Mountain View, CA, USA) combined with the pLVX-Puro lentiviral expression vector (Clontech) was used to generate lentiviruses in HEK293T cells, and after harvesting and filtration through a 0.45 µm cellulose acetate filter, viruses were stored at −80 °C. To transform RPE1 cells, 1–2 mL virus-containing media was added to 50%–70% confluency RPE1 cells in 10 cm dishes. Virus was removed by washing after 48 h, and stable cells were selected by incubating cells in 5 µg/mL puromycin. Transient transfection of plasmids into RPE1 cells was carried out using GeneJuice (Novagen, Billerica, MA, USA) according to the manufacturer’s instructions.

### 4.4. siRNA Library and Transfection

The siRNA SMARTpool library used in the initial screens was purchased from Dharmacon (Thermo Scientific, Paisley, UK) with the exception of the STX17 oligonucleotides that were from Invitrogen (gene identifiers and catalog numbers are shown in Table 1). For the autophagy siRNA screen in 96-well glass bottom plates (MatTek Corp., Ashland, MA, USA), a calcium phosphate reverse knock down strategy was used. Cells were plated in oligonucleotide (20 μM) containing CaCl_2_ mix (equal volumes of 0.25 M CaCl_2_ and BBS (50 mM BES, 280 mM NaCl, 1.5 mM Na_2_PO_4_ at a pH of 6.95)). The cells were left in siRNA overnight in 3% CO_2_ in a humidified 37 °C incubator before fresh media was added. Validation siRNA silencing experiments were carried out using a standard, forward calcium phosphate knock down approach. For the mitophagy screen a lipid-based reverse transfection protocol was employed, using Lipofectamine^®^2000 transfection reagent (Life Technologies, Paisley, UK). The following siRNA oligonucleotides were obtained from MWG Eurofins, unless stated otherwise: non-targeting control (CGU ACG CGG AAU ACU UCG A); RISC-free (Dharmacon siGENOME^®^: D-001220-01, Lafayette, CO, USA); SIGMAR1a (GAG CUG GCC UUC UCU CGU C); SIGMAR1b (GAA UGC GGG UGG CUG GAU G); SIGMAR1c (GCG AAG AGA UAG CGC AGU U); SIGMAR1d (CCG AGU AUG UGC UGC UCU U); SIGMAR1 UTR (GUC AGC GUC UUC CAU UCC A); ATG12 (CCA UCC AAG GAC UCA UUG A); ATG3 (CAC AGG UAU UAC AGG AAU A); DHC1 (ACA UCA ACA UAG ACA UUC ATT); MCU (Dharmacon SMARTpool: M-015519-00, Lafayette, CO, USA).

### 4.5. Microscopy

Fixed and live-cell imaging was performed using an Olympus IX-71 inverted microscope with a 60× Uplan Flourite objective lens (0.65–1.25 NA, oil). Images were obtained using a CoolSNAP HQ2 CCD camera (Photometrics, Tuscon, AZ, USA) driven by MetaMorph software (Molecular Devices, Sunnyvale, CA, USA). For live-cell imaging, cells were grown on 3 cm culture dishes containing glass inserts (MatTek Corp.). For the siRNA screens on 96-well plates, media was first removed by upturning the plates on to a piece of tissue paper, before 100 µL 3.7% formaldehyde solution in cell culture media was added to each well. Cells were then washed and permeabilised as required and processed for immunofluorescence imaging. To image 96-well plates, a 40× Uplan Flourite objective lens (0.75 NA, dry) was used and at least 7 fields of view per well were acquired for analysis, and conditions associated with retention of mitochondrial content across individual fields were scored as positive. For validation of mitophagy using individual siRNAs and siRNA combinations, the percentage of cells having >5 clearly discernable mitochondria following antibody staining was calculated. To count autophagosome and assembly site puncta, images were filtered using the TopHat algorithm, gating for circular objects of up to 5 pixels. Thresholds were set manually for each experimental data set, and the MetaMorph “count cells” plugin was used to automatically count the number of puncta/field.
